# Acknowledgement of manuscript reviewers the underappreciated contributors

**DOI:** 10.1186/s12986-016-0078-x

**Published:** 2016-03-02

**Authors:** Ahmed Bakillah, M Mahmood Hussain

**Affiliations:** SUNY Downstate Medical Center, New York, USA

## Abstract

We and the Editorial Board sincerely acknowledge and thank all the reviewers for their active participation and contribution during 2015 (Volume 12). We greatly appreciate their dedication and behind the scenes contribution. It is largely due to their support and expertise that we have been able to publish high standard manuscripts. We would also like to thank authors for choosing *Nutrition & Metabolism* and contributing their cherished work to this journal.

**A. Schlegel**

United States of America

**A. A. Tinkov**

Russian Federation

**Abdalla El-mowafy**

Egypt

**Ahmed Bakillah**

United States of America

**Alan Ducatman**

United States of America

**Alan Hayes**

Australia

**Altan Onat**

Turkey

**Amit Tyagi**

India

**Amy Bentley**

United States of America

**Anna Litwic**

United Kingdom

**Anssi Manninen**

Finland

**Antonio Camargo**

Spain

**Arpita Basu**

United States of America

**B. Scolnick**

United States of America

**B. A. Patel**

United Kingdom

**Balachandar Nedumaran**

United States of America

**Bettina Pfleiderer**

Germany

**Bill Lands**

United States of America

**Brian Bennett**

United States of America

**Bruce Ames**

United States of America

**Bruno G. Oertel**

Germany

**C. A. Cooper**

United States of America

**Carrie Durward**

United States of America

**Chen Zheng**

United States of America

**Chiara Ferrario**

Italy

**Chris McEntyre**

New Zealand

**Christine Metz**

United States of America

**Christos Derdemezis**

Greece

**C-Y. Oliver Chen**

United States of America

**D. Chartoumpekis**

United States of America

**Damien Belobrajdic**

Australia

**Daniel Konrad**

Switzerland

**Darius Batulevicius**

Lithuania

**D. C. Damasceno**

Brazil

**D. D. Leonidas**

Greece

**Ddo N. Marreiro**

Brazil

**D. L. Williamson**

United States of America

**D. M. Camera**

Australia

**Donald Layman**

United States of America

**Dong Cheng**

United States of America

**Donghui Zhang**

United States of America

**Douglas Kalman**

United States of America

**E. James Squires**

Canada

**E. Saad**

Egypt

**E. Zambrano**

Mexico

**EI Magalhães**

Brazil

**Eirik Degerud**

Norway

**Elizabeth Beierle**

United States of America

**E. M. Pereira**

Brazil

**Everson Nunes**

Brazil

**Fabio Lira**

Brazil

**Fabrice Tranchida**

France

**Fareeba Sheedfar**

Netherlands

**Fasika Tedla**

United States of America

**Federico Salomone**

Italy

**Fei Fang**

United States of America

**Felipe Vadillo-Ortega**

Mexico

**Forrest Nielsen**

United States of America

**Fotios Iliadis**

Greece

**G. Jamar**

Brazil

**G. Łysiak**

Poland

**Gary Lopaschuk**

Canada

**Gm Dallinga-Thie**

Netherlands

**Gustavo Pimentel**

Brazil

**H. Kaji**

Japan

**H. Mirmiranpour**

Islamic Republic of Iran

**H. Yeger**

Canada

**Hans Degens**

United Kingdom

**Hossam Elnoamany**

Egypt

**J. Farinha**

Brazil

**J. Liang**

China

**J. Palmfeldt**

Denmark

**J. Pepe**

Italy

**Jahangir Iqbal**

United States of America

**Jamie Baum**

United States of America

**Jan Palmblad**

Sweden

**Javier Montalvo**

Mexico

**J. H. Lee**

United States of America

**J. J. DiNicolantonio**

United States of America

**J. J. Worthington**

United Kingdom

**J. L. Burkhead**

United States of America

**J. L. Hernández**

Spain

**Johanna Apro**

Sweden

**Jonathan Steven Alexander**

United States of America

**Jong Rho**

Canada

**Joost Willebrords**

Belgium

**Jørgen Jensen**

Norway

**Jose Antonio**

United States of America

**Jose Donato Jr.**

United States of America

**Joshua Meidenbauer**

United States of America

**J. R. Trevithick**

Canada

**Jt Jr Nickels**

United States of America

**Juei-Tang Cheng**

Taiwan

**Jung-Su Chang**

Taiwan

**J. Y. Chang**

Taiwan

**K. Jarukamjorn**

Thailand

**K. Roy**

Australia

**Katherine Crew**

United States of America

**Katherine Veras**

Brazil

**Ke Li**

United States of America

**Kevin Maki**

United States of America

**Kezhi Dai**

United States of America

**Kezhi Dai**

United States of America

**Kiatlyn Liu**

United States of America

**KM Weinberge**

United Kingdom

**Kun-Ruey Shieh**

Taiwan

**L. Duplomb**

France

**Larry Rudel**

United States of America

**Latha Devi**

United States of America

**L. D. Kong**

China

**Lei Hao**

United States of America

**Leng Huat Foo**

Malaysia

**Leonardina Ciccarelli**

Italy

**Lharbi Dridi**

Canada

**Lianfeng Wu**

United States of America

**Ling Yang**

United States of America

**Liye Zhou**

United States of America

**L. R. Brunham**

Canada

**Luis Salazar-Olivo**

Mexico

**Lydie Combaret**

France

**M. Alemany**

Spain

**M. Böhm**

Australia

**M. Dixit**

India

**M. Fex**

Sweden

**M. Fisberg**

Brazil

**M. Horiuchi**

Japan

**M. Yamato**

Japan

**Mahmood Hussain**

United States of America

**Marco Manca**

Netherlands

**Maria Victoria Garcia-Mediavilla**

Spain

**Mark Herman**

United States of America

**Martha Belury**

United States of America

**Martha Rodriguez-Moran**

Mexico

**Martin Burtscher**

Austria

**Martin Joyce-Brady**

United States of America

**Mary Honors**

United States of America

**Masanori Yoshizumi**

Japan

**Matthew Cannon**

United States of America

**Matthew Picklo**

United States of America

**M. C. Gomes-Marcondes**

Brazil

**Meghan Walsh**

United States of America

**Melissa Benton**

United States of America

**Mengwei Zang**

United States of America

**M. H. Sharawy**

Egypt

**M'hamed Bentourkia**

Canada

**Min Yang**

China

**Mingming Gao**

United States of America

**M. J. Keenan**

United States of America

**M. J. Morris**

Australia

**M. J. Rowling**

United States of America

**Mohammed Al-Gayyar**

Egypt

**Mostafa Waly**

Oman

**M. S. Fawzy**

Egypt

**N. Keleku-Lukwete**

Japan

**Neha Garg**

United States of America

**Nicholas Davidson**

United States of America

**N. J. Kellow**

Australia

**O. D. Rangel-Huerta**

Spain

**P. Bhattacharyya**

Puerto Rico

**P. Liu**

China

**Palanikumar Manoharan**

United States of America

**P. C. Lisboa**

Brazil

**P. D. Portales-Pérez**

Mexico

**Philip Calder**

United Kingdom

**Philippe Costet**

United States of America

**Pietro Ragonesi**

Italy

**P. K. Dudeja**

United States of America

**P. O. Prada**

Brazil

**Qiang Fu**

China

**Qiang Li**

United States of America

**R. Fujiwara**

Japan

**R. Vij**

India

**R. A. Gómez-Díaz**

Mexico

**Rai Ajit Srivastava**

United States of America

**Randolph Matthews**

United States of America

**Rebecca MacPherson**

Canada

**Ren Xu**

United States of America

**Robert Harris**

United States of America

**Rosa Luciano**

Italy

**Rosana Pagano**

Brazil

**Ryuichi Kawamoto**

Japan

**S. Chung**

United States of America

**S. Eghbalsaied**

Islamic Republic of Iran

**S. Klaus**

Germany

**S. Reddi**

India

**S. Sanchez-Enriquez**

Spain

**S. A. Kumar**

Australia

**Saihan Borghjid**

United States of America

**Salman Azhar**

United States of America

**Samy McFarlane**

United States of America

**Sandra Peters**

Canada

**Sara Auharek**

Brazil

**Selma Liberato**

Australia

**S. H. Hyun**

Democratic People’s Repubilc of Korea

**Shanshan Pei**

United States of America

**Shi Sun**

United States of America

**Silvia Savastano**

Italy

**Srinidi Mohan**

United States of America

**Stanley Hazen**

United States of America

**Stephane Walrand**

France

**Stephanie Chung**

United States of America

**Streamson Chua**

United States of America

**Sunil Panchal**

Australia

**Susan Arthur**

United States of America

**Sushma Reddy Gundala**

United States of America

**T. Luo**

China

**T. A. Wihastuti**

Indonesia

**Takeshi Hase**

Japan

**Tamás Decsi**

Hungary

**Tatjana Stojakovic**

Austria

**Thad Rosenberger**

United States of America

**Thirupathi Muthusamy**

United States of America

**Tia Rains**

United States of America

**T. J. Huppert**

United States of America

**T. K. Lee**

Australia

**T. L. Marin**

United States of America

**Toshiaki Tamaki**

Japan

**Tracy Anthony**

United States of America

**Tyler Barker**

United States of America

**Uwe Tietge**

Netherlands

**V. Velarde**

Chile

**Vasilios Athyros**

Greece

**Vathsala Mohan**

New Zealand

**Venu Velagapudi**

United States of America

**Vera Mazurak**

Canada

**Vidya Velagapudi**

Finland

**Vikas Kumar**

India

**W. Wang**

China

**W. Zhang**

United States of America

**Wayne Grody**

United States of America

**Wei Song**

United States of America

**W. M. Xu**

China

**Wolfram Doehner**

Germany

**X. Prieur**

France

**Xi Li**

China

**Xian-Cheng Jiang**

United States of America

**Xiaoyue Pan**

United States of America

**Xin Bi**

United States of America

**Xinyin Jiang**

United States of America

**Xueying Chen**

China

**Xueying Chen**

United States of America

**Y. Kawano**

Japan

**Yang Zhihong**

United States of America

**Yiyi Ma**

United States of America

**Yong Hu**

United States of America

**Yong Zhu**

United States of America

**Y. T. Kuo**

Japan

**Yvonne Fierz**

Switzerland

**Z. Ning**

China

**Zehra Ilke Akyildiz**

Turkey

**Zengying Wu**

United States of America

